# Alteration of Sequence Specificity of the Type IIS Restriction Endonuclease BtsI

**DOI:** 10.1371/journal.pone.0011787

**Published:** 2010-07-26

**Authors:** Shengxi Guan, Aine Blanchard, Penghua Zhang, Zhenyu Zhu

**Affiliations:** New England Biolabs, Inc., Ipswich, Massachusetts, United States of America; University Paris Diderot-Paris 7, France

## Abstract

The Type IIS restriction endonuclease BtsI recognizes and digests at GCAGTG(2/0). It comprises two subunits: BtsIA and BtsIB. The BtsIB subunit contains the recognition domain, one catalytic domain for bottom strand nicking and part of the catalytic domain for the top strand nicking. BtsIA has the rest of the catalytic domain that is responsible for the DNA top strand nicking. BtsIA alone has no activity unless it mixes with BtsIB to reconstitute the BtsI activity. During characterization of the enzyme, we identified a BtsIB mutant R119A found to have a different digestion pattern from the wild type BtsI. After characterization, we found that BtsIB(R119A) is a novel restriction enzyme with a previously unreported recognition sequence CAGTG(2/0), which is named as BtsI-1. Compared with wild type BtsI, BtsI-1 showed different relative activities in NEB restriction enzyme reaction buffers NEB1, NEB2, NEB3 and NEB4 and less star activity. Similar to the wild type BtsIB subunit, the BtsI-1 B subunit alone can act as a bottom nicking enzyme recognizing CAGTG(-/0). This is the first successful case of a specificity change among this restriction endonuclease type.

## Introduction

Restriction-Modification (R-M) systems mostly occur naturally in bacteria and serve as defensive systems against invading DNA, particularly bacteriophages. The restriction endonuclease digests invading DNA while the DNA methyltransferase component(s) (cognate methyltransferase(s)) modifies the host genome and protects the host DNA from restriction endonuclease digestion [1]. There are four groups of restriction endonucleases (REases): Type I, Type II, Type III and Type IV [2]. Among the four different Types, Type II REases have been widely studied and used as tools in molecular biology, largely due to their well-defined recognition and cleavage sites. So far, more than 3800 Type II REases with 299 distinct specificities have been characterized biochemically or genetically [3]. Among the subdivisions of Type II REases, the Type IIS are unique in having asymmetric recognition sequences [2].

BtsI (Genbank: CS808224.1), a Type IIS REase, was isolated from *Bacillus thermoglucosidasius* (Pan, X.S., Morgan, R. unpublished observation). BtsI REase consists of two subunits BtsIA and BtsIB, and the modification system also contains two methyltransferases (M1.BtsI and M2.BtsI) [4]. Each methyltransferase modifies one strand of the recognition sequence. BtsIB, the large subunit, contains a DNA recognition domain and a catalytic domain that can act naturally as a bottom strand nicking enzyme. The recognition sequence is written as GCAGTG(-/0), which means that BtsIB does not cut the top strand and cuts right after the recognition sequence on the bottom strand. BtsIA, the small subunit, contains part of the catalytic domain responsible for top-strand cleavage. BtsIA alone is inactive. Only when BtsIA is combined with BtsIB do they form a restriction endonuclease capable of double-strand cleavage (GCAGTG(2/0))[4]. The cleavage sequence is GCAGTG(2/0), which means that BtsI cuts 2 nt after the recognition sequence on the top strand and right after the recognition sequence on the bottom strand.

Numerous efforts have been made to engineer REases for altered specificity but with rather few successful examples. Several groups reported success in altering the specificity of Type IIG REases. For example, Eco57I (CTGAAG(16/14)) has been engineered to Eco57MI (CTGRAG(16/14)) by random mutagenesis followed by challenges with Eco57I (CTGAAG(16/14) and GsuI(CTGGAG(16/14)) [5]. AloI((7/12-13)GAACNNNNNNTCC(12-13/7)) a Type IIG-like REase with two recognition domains, can acquire new recognition and digestion specificities by domain swapping with another similar Type IIG REase [6]. One major cluster of successful examples is from MmeI(TCCRAC(20/18) or TCCRAC(21/19))-like Type IIG REases. By analyzing the amino acid sequence of a group of closely related REases, specificity can be rationally altered by changing the corresponding amino acid residues in the protein [7]. All of these successful examples are Type IIG REases, in which endonuclease, methylase, and specificity domains are fused into a single polypeptide chain.

Other than Type IIG REases, there are reports of incomplete specificity changes with certain preference for one particular sequence. For example, some mutants of BsoBI change specificity from C/YCGRG to prefer C/CCGGG, C/TCGGG or C/TCGAG [8]. BstYI has been engineered to favor A/GATCT than R/GATCY [9]. HincII (Q138F) shows a stronger preference for GTT/AAC and GTC/GAC than wild type enzyme [10]. However, engineering the catalytic domain is relatively easier than engineering the recognition sequence. One successful example is the engineering of several nicking enzymes including Nt.AlwI[11], Nt.BbvCI, Nb.BbvCI[12], [13], Nt.MlyI[14], Nt.BspQI[15], and Nt.BsmAI[16].

Here, we describe a BtsIB mutant (BtsIB:R119A) with altered specificity. After careful examination, the new specificity is one base pair shorter than the wild type enzyme. The new recognition sequence is CAGTG(-/0) compared with the wild type GCAGTG(-/0). When combined with the A subunit, BtsIB (R119A) can recognize and cleave the following sequence CAGTG(2/0). This specificity change is complete and shows little preference for any other specific sequences. To the best of our knowledge, this essentially complete specificity change of BtsI is the first successful example of such in REases other than Type IIG. The new enzyme BtsI(B:R119A) was named as BtsI-1. Correspondingly, the BtsIB(R119A) was named as Nb.BtsI-1.

## Results

### Discovery of a BtsIB variant with different digestion patterns

**Figure 1:**
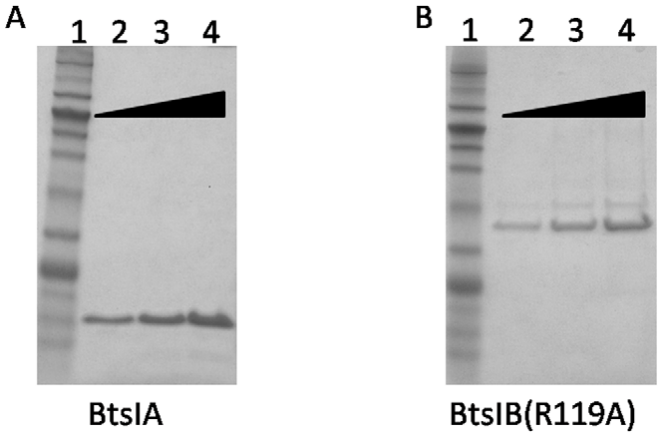
Purification of BtsIA and BtsIB(R119A) with IMPACT system. 1 µl (lane 2), 2 µl (lane 3) and 4 µl (lane 4) of eluted BtsIA protein (A) or BtsIB(R119A) (B) were resolved by SDS-PAGE and stained with Coomassie Blue. Lane 1: protein ladder (NEB #P7711S)

**Figure 2:**
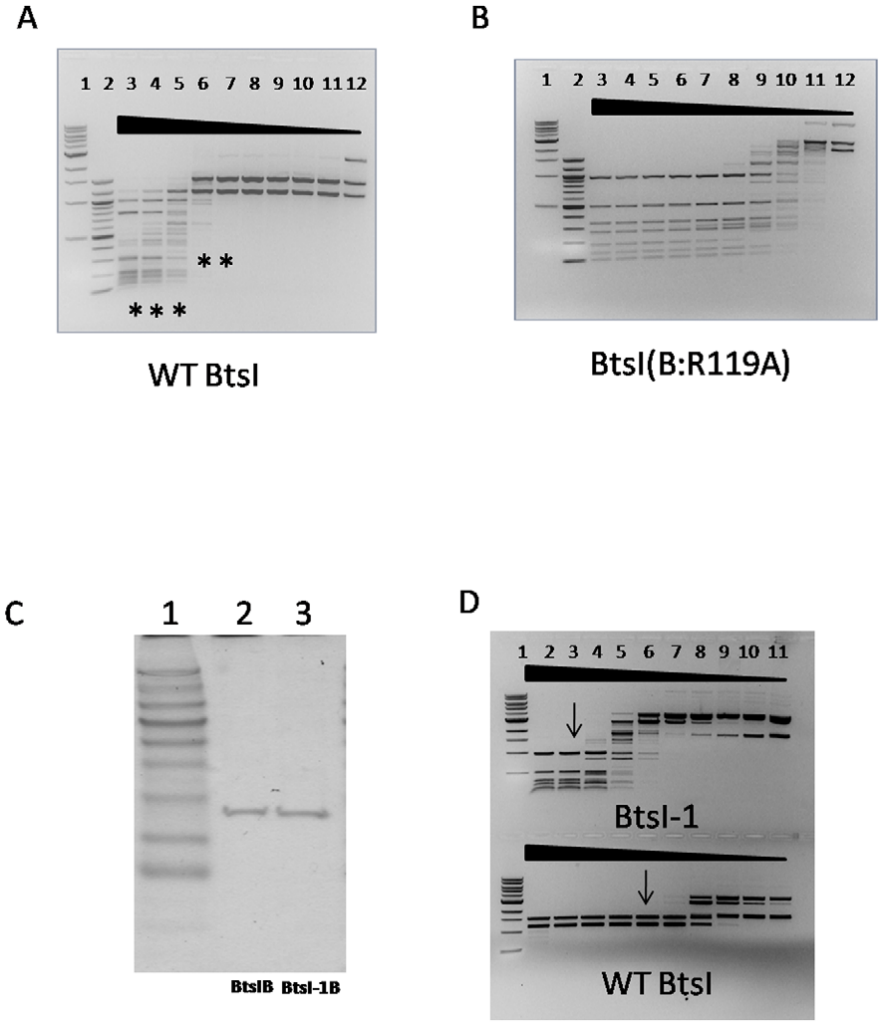
BtsIB(R119A) has different cutting patterns and less specific activity compared with wild type BtsIB. A: A 2-fold serial dilution of wild type BtsIB (∼28 pmol) was mixed with a constant concentration of excess BtsIA (∼43 pmol) and used to digest pUC19 (0.6 µg) in NEB4 at 55°C for 1 hour (lane 3–12). Symbol stars (*) from lanes 3–7 indicate that star activities appear under these BtsI concentrations. Lanes 8–10 show complete digestion patterns. Lanes 11–12 show incomplete digestion pattern. B: A 2-fold serial dilution of BtsIB(R119A) (∼20 pmol) was mixed with a constant concentration of excess BtsIA (∼43 pmol) and used to digest pUC19 (0.6 µg) in NEB4 at 55°C for 1 hour (lanes 3–12). Lanes 3–7 show complete digestion patterns, whereas lanes 8–12 show incomplete digestion patterns. Lane 1: 1 kb DNA Ladder (NEB #N3232); Lane 2: 100 bp DNA Ladder (NEB N3231). C: The same concentration of BtsIB and BtsI-1B (∼1.8 µM) were resolved by SDS-PAGE and stained with Coomassie Blue. D: The same amount of BtsIB and BtsI-1B (∼3.6 pmol) were mixed with BtsIA (∼3.6 pmol) at the molar ratio of 1∶1 and then were serially diluted by 2-fold to digest pUC19 (0.6 µg) in NEB4 at 55°C for 1 hour. Arrows indicates the lane with minimal amount of protein for complete digestion.

During the process of BtsIB subunit mutagenesis aiming to investigate the effect of a single amino acid change on its protein function, one BtsIB mutant (R119A) was found to have a different digestion pattern from the wild type BtsIB. To avoid contamination from the host bacteria, we used the IMPACT system to purify BtsIB(R119A) and BtsIA to near homogeneity, as shown in Figure 1. The concentration for BtsIA is around 0.80 mg/ml or 43 µM and the concentration for BtsIB(R119A) is about 0.37 mg/ml or 9.8 µM. Wild type BtsI can cut pUC19 into three fragments: 1.5 kb, 1.1 kb and 20 bp, as shown in Figure 2A (lane 8–11). The 20 bp fragment was too small to be detected in the 1% agarose gel. At high concentrations, wild type BtsI showed severe star activity (lane 3–7). Interestingly, BtsI(B:R119A) cleavage of pUC19 produces a larger number of small fragments than the wild type BtsI does, as shown in Figure 2B. These fragments remained unchanged even as protein concentration increased, suggesting that these fragments represented a complete digestion. Also, the digestion patterns were different from the star activity bands generated by the wild type BtsI (Fig. 2A lane 3 vs. Fig. 2B lane 3). From these results, we concluded that BtsI(B:R119A) might have a specific and shorter recognition sequence than WT. Therefore, we designated BtsI(B:R119A) as BtsI-1. Correspondingly, BtsIB(R119A) is named as BtsI-1B.

To compare the specific activity of BtsIB and BtsI-1B, we used same amount of each enzyme (Fig. 2C) to digest pUC19. These enzymes were first mixed with BtsIA subunit at the molar ratio of 1∶1, performed 2-fold serial dilution and then digested pUC19. As shown in Fig. 2D, the specific activity of BtsI-1 is 1/8 of BtsI WT. When compared on a pair of oligos with only one site for either BtsI WT or BtsI-1, the activity of BtsI-1 is still 1/8 of that of BtsI WT (data not shown).

### Characterizing recognition sequences and cutting sites of BtsI-1

**Figure 3:**
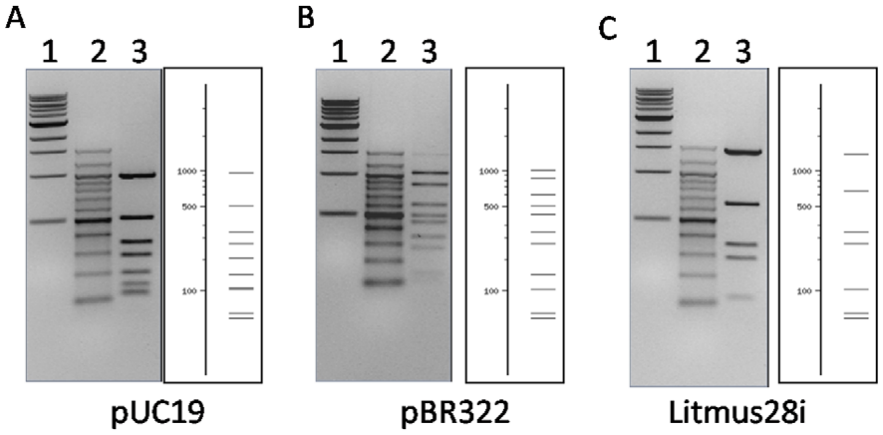
The digestion patterns of BtsI-1 on different substrates: 1 µl of BtsI-1B (∼1.8 µM) was mixed with 1 µl of BtsIA and then digested 0.6 µg pUC19 (A), pBR322 (B) and Litmus28i (C) in NEB4 at 55°C for 1 hour. In each figure, Lane 3 in the left panel is the actual digestion pattern and the right panel is the theoretical digestion pattern generated by NEBcutter V2.0. Lane 1: 1 Kb DNA Ladder (NEB #N3232); Lane 2: 100 bp DNA Ladder (NEB #N3231).

To identify the recognition sequence of BtsI-1, we analyzed the pUC19 fragments digested by BtsI-1with REBpredictor [17]. The program predicted that BtsI-1 recognizes the sequence “CAGTG”. To confirm our prediction, BtsI-1was used to digest different plasmids including pUC19, pBR322 and Litmus28i. As shown in Figure 3, all digestion patterns on the left appeared to be the same as the theoretical digestion patterns on the right as created by NEBcutter V2.0. Compared with the BtsI recognition sequence “GCAGTG”, BtsI-1 no longer recognizes the first “G” which strongly suggests that Arg119 plays an important role for the first “G” recognition.

**Figure 4:**
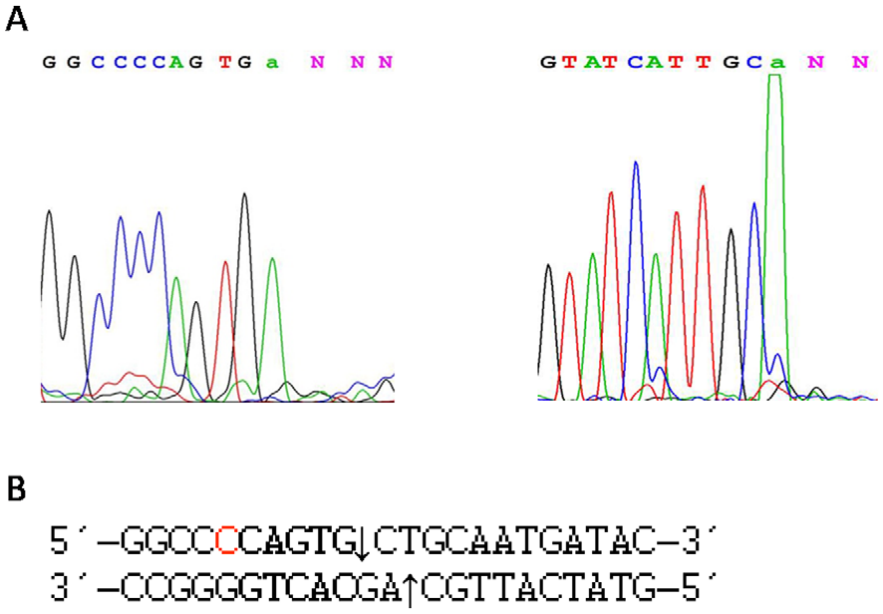
Run-off sequencing to determine the actual cutting site of BtsI-1. A: pUC19 (0.6 µg) was digested by BtsI-1(∼1.8 pmol) and subjected to run-off sequencing with two flanking primers for both directions. The drop in the peak reflects where the polymerase runs off the template at the nicked sites. The terminal “a” is automatically added to the end by the polymerase during sequencing. B: The sequencing data were aligned with pUC19 sequence. The nicking sites are indicated by arrows on both strands.

To determine the cutting sites of BtsI-1, we cut pUC19 with BtsI-1 for 1 hour and subjected the substrate to run-off sequencing. Primers flanking one recognition sequence allowed sequencing from both directions, and the resulting sequences were aligned with pUC19 (Figure 4). After alignment, the cutting site for BtsI-1 is (C)CAGTG(2/0), which is not recognized by wild type BtsI, whose cutting site is GCAGTG(2/0). However, the 3′ 2-base overhang after digestion remains unchanged.

### The effect of other residues at position 119 of BtsIB

**Figure 5:**
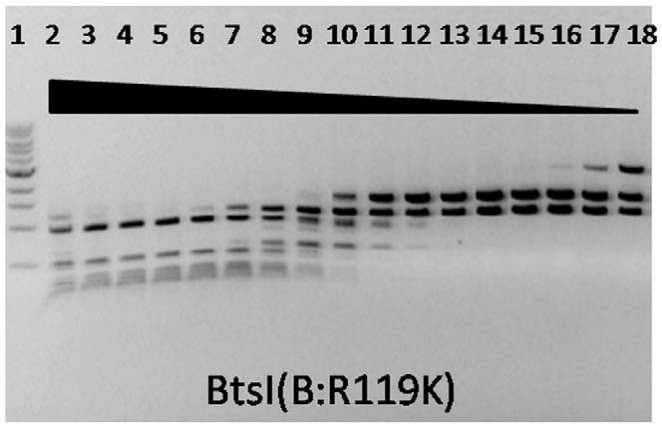
Specific activity of BtsI(B:R119K) mutant on pUC19. BtsIB(R119K) was partially purified, 2-fold serially diluted and mixed with constant amount of BtsIA (∼43 pmol) to digest pUC19 (0.6 µg) in NEB4 at 55°C for 1 hour. Lane 1: 1 kb DNA ladder.

We explored whether substitution of other amino acid residues at position 119 of B subunit would also alter BtsI sequence recognition. R119G, R119H, R119I, R119L, R119M, R119N, R119S, R119T, R119V, R119W and R119Y showed cleavage patterns similar to the R119A mutant. It is interesting to note that BtsI(B:R119K) retained the cleavage specificity of wild type BtsI at low concentration (Fig. 5, lane 13–16). However, as the concentration increased, the cutting pattern converted to that of BtsI-1 (Fig. 5, lane 2–12). All other mutants, including R119C, R119D, R119E, R119F, R119P, and R119Q, did not have significant activity (data not shown).

### BtsI-1 displays different relative activities in NEB1, NEB2, NEB3 and NEB4

**Figure 6:**
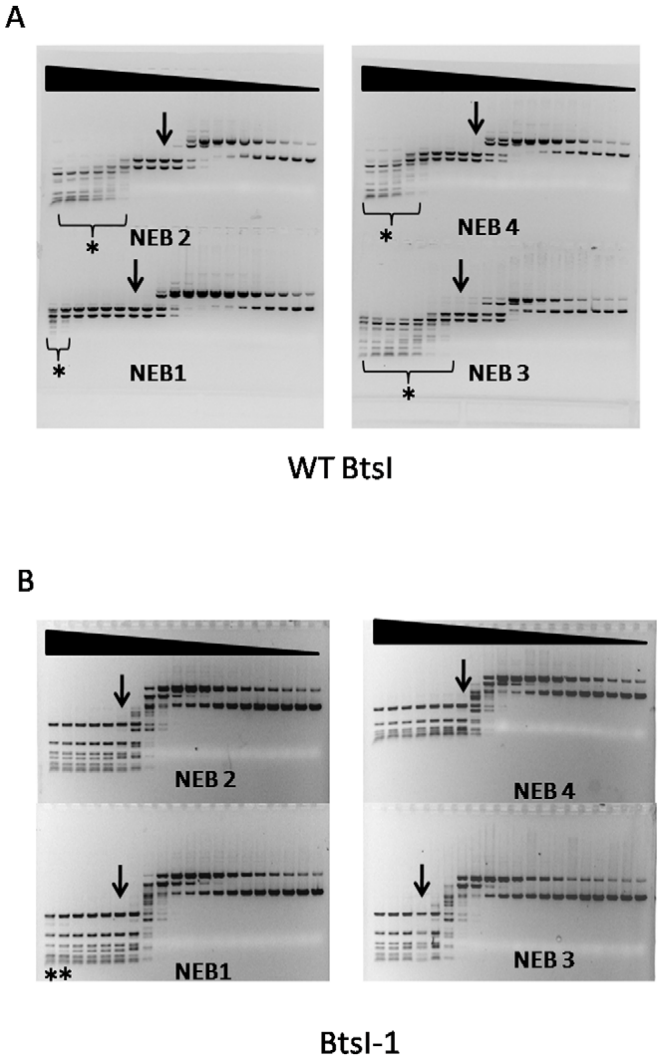
Comparison of relative activity between BtsI and BtsI-1 in NEB1, NEB2, NEB3 and NEB4. Wild type BtsIB (∼28 pmol) (A) or BtsIB-1 (20 pmol) (B) was mixed with same amount of BtsIA, 2-fold serially diluted and used to digest pUC19 (0.6 µg) for 1 hour at 55°C in NEB1, NEB2, NEB3 or NEB4. Stars (*) indicate that star activities appeared at these concentrations. Arrows indicate the lane with complete digestion pattern with minimal protein concentration.

Compared to wild type BtsI, BtsI-1 displays different relative activities in the NEB buffer system. As shown in Figure 6A, wild type BtsI has the highest activity in NEB2 and NEB4. In comparison to activity in these buffers, BtsI activity in NEB1 and NEB3 was only 25% and 50% respectively. In contrast, the highest BtsI-1 activity was observed in NEB4. In NEB1, NEB2 and NEB3, BtsI-1 had 50%, 50% and 12.5% activity, respectively (Figure 6B). It is interesting to note that relative activity drops dramatically in NEB3, presumably due to salt inhibition.

### BtsI-1 has less star activity than BtsI

Wild type BtsI has severe star activity whereas BtsI-1 has less star activity (Figure 6). To quantify the star activity, we calculated the Fidelity Index (FI) [18]. Wild type BtsI has FIs of only 8, 2, 2 and 4 in NEB1, NEB2, NEB3 and NEB4, respectively (Figure 6A). However, BtsI-1 has FIs of 8, ≥32, ≥8 and ≥64 in the same buffer series (Figure 6B). Larger FIs in each buffer means that the star activity of BtsI-1 is less than that of BtsI.

### BtsI-1B acts as a bottom strand nicking enzyme

**Figure 7:**
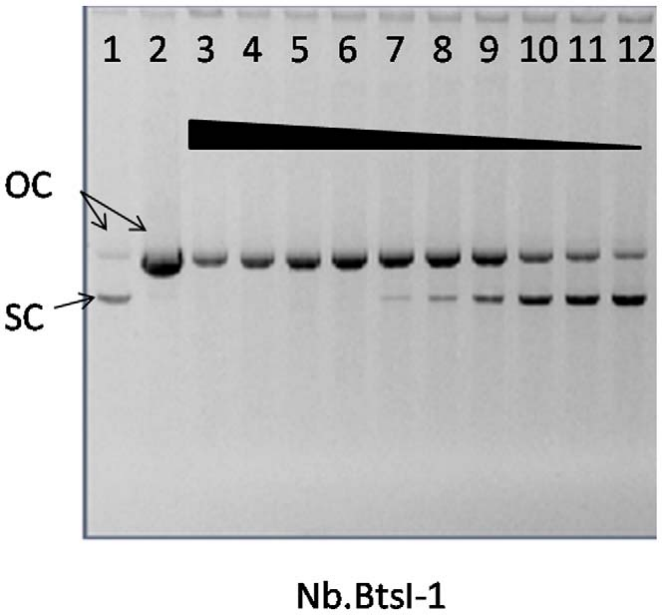
Nicking activity of Nb.BtsI-1 on substrate phiX174 RF I. A 2-fold serial dilution of Nb.BtsI-I was used to digest phiX174 RF I at 55°C for 1 hour (lane 3–20). Lane 1 is uncut phiX174 RF I. Lane 2 is uncut phiX174 RF II (Nicked, circular form of phiX174). OC: open circular; SC: super coil.

The isolated wild type BtsIB alone naturally acts as a bottom strand nicking enzyme with the cutting sequence GCAGTG(-/0). Considering this, we digested phiX174 RFI with BtsI-1B. As shown in Figure 7, BtsI-1B nicks phiX174 RFI to form open circular DNA. Run-off sequencing of the fragments suggested that BtsI-1B also acts as a bottom nicking enzyme with the cutting sequence CAGTG(-/0) (data not shown). Thus, we name BtsI-1B as Nb.BtsI-1.

## Discussion

Engineering new REase specificities has been limited mainly due to two reasons: 1) the complexity and extended nature of the binding interaction between REases and DNA: ∼15-20 hydrogen bonds, numerous van der Waals contacts [19]; 2) Inability to produce the altered REase in expression systems due to the lack of proper protection (mainly proper methyltransferase). So far most of the successful specificity-changed REases are Type IIG REases, due to the fact that Type IIG REases have both restriction endonuclease activity and methylase activity on the same protein, sharing the same recognition sequences. In Type IIG REases, this allows the expression strain to always be protected. However, for other REases, the restriction endonuclease activity and the methyltransferase activity are carried out by two different enzymes. Once the recognition sequences of REases change to those beyond the protection of methyltransferase, the host strain will be killed. The reason that BtsI-1 can survive after the specificity change is because we expressed the BtsIA and BtsIB separately. The BtsIB variant only nicks the unprotected hosts genomic DNA, which is less toxic to the host than complete double strand cleavage. The strain containing pUC19BtsI-1B and pACYCBtsIM1M2 is not stable, indicating that nicking BtsI-1B is not fully protected by the two methylases M1.BtsI and M2.BtsI.To express BtsI-1B, the tightly controlled vector pBAD241was required. With this pBAD241 vector, the expression level of the BtsI-1B can reach 50,000 units for each gram of cells.

The typical PD-EXK motif of BtsIB is at P57, D58 and E72. R119 is distant from those residues on the polypeptide chain, yet it still can affect the catalytic site and apparently is involved in the recognition of the first G in GCAGTG; however, there is no crystal structure currently available, and there are no other restriction enzymes that recognize HCAGTG (H = A or C or T) to offer insight as to how R119 recognizes the first G. Crystallization of BtsI and BtsI-1 with DNA would be an essential step for further understanding.

The star activity for wild type BtsI is so severe that it is impossible to overexpress BtsIA and BtsIB subunits in the same strain even with M1.BtsI and M2.BtsI protection. However, it is interesting to note that BtsI-1 has decreased star activity. BtsI-1 is the first REase other than Type IIG with a complete specificity change. It provides a new direction for future research into changing the specificity of REases. As specificity engineering cases accumulate, understanding the mechanisms of the specificity determination will increase.

## Materials and Methods

### Reagents, strains and vectors

All reagents, strains and vectors, unless stated otherwise, were from New England Biolabs, Inc. (Ipswich, MA, USA)

### Sub-cloning, Expression and Purification of BtsI.A and BtsI.B mutant

BtsIA and BtsIB genes were cloned in pUC19 and BtsIM1M2 was cloned in pACYC184, as previously described [4]. To obtain pure BtsIA or BtsIB(R119A) subunits, the IMPACT (Intein Medicated Purification with an Affinity Chitin-binding Tag) system (New England Biolabs) was used for one-step purification. Briefly, the BtsIA gene was sub-cloned into the pTXB1 vector, which was then transformed into competent *E. coli* strain with the methylase protection ER2566(pACYC184-Bts1M1M2) containing the T7 RNA polymerase, controlled by the *lac* operon. The bacteria were induced by IPTG for protein expression and lysed via sonication. After centrifugation, the supernatant was loaded onto a chitin column, which was equilibrated with chitin column buffer (20 mM Tris-HCl, pH 8.5, 500 mM NaCl). After washing with the same column buffer, the column was incubated with Cleavage Buffer (20 mM Tris-HCl, pH 8.5, 500 mM NaCl and 50 mM DTT) at 4°C overnight. The protein was then eluted. The pure BtsIA protein was dialyzed against the storage buffer (10 mM Tris-HCl pH 7.4, 0.1 mM EDTA, 1 mM DTT, 50 mM KCl and 50% Glycerol).

For BtsIB(R119A), the gene was fused with Mxe intein at C-terminal and cloned into pBAD241, a derivative vector from pBAD24, with some changes in the multiple cloning sites. After expression, the BtsIB(R119A) was purified with the same procedure as the BtsIA. For BtsIB(R119K), the protein was expressed in ER2984 and partially purified through Heparin Column (GE Healthcare).

### Run-off sequencing and DNA sequencing

All sequencing procedures were performed as previously described[16].

### Site-directed Mutagenesis

By using site-directed mutagenesis with Vent polymerase and inverse PCR, charged or polar residues (including His, Lys, Arg, Asp, Glu, Ser, Thr, Gln, and Asn) were individually mutated to Ala one residue at a time, and each Tyr residue was mutated to Phe. After DpnI digestion, the PCR products carrying the mutated sequences were transformed into a methyltransferase-protected (M1.BtsI and M2.BtsI) bacterial strain *E. coli* ER2984(pACYC184-BtsIM1M2) for expression.

### Activity assay

To examine the effect of the mutated BtsIB, 2 µl crude extract or purified protein containing the mutated subunit was mixed with 1 µl purified BtsIA subunit, either at molar ratio of 1∶1 or with constant excess amount of A subunit, to reconstitute the full endonuclease activity of each BtsI mutant. The mixture of total 30 µl reaction was incubated with pUC19 (0.6 µg) in NEB1, NEB2, NEB3 or NEB4 for 1 hour at 55°C; then, the reaction was resolved on an agarose gel. The composition of 4 different reaction buffers as following: NEB1∶ 10 mM Bis Tris Propane-HCl, 10 mM MgCl_2_, 1 mM dithiothreitol (pH 7.9 @ 25°C); NEB2∶ 50 mM NaCl, 10 mM Tris-HCl, 10 mM MgCl_2_, 1 mM dithiothreitol (pH 7.9 @ 25°C); NEB3∶ 100 mM NaCl, 50 mM Tris-HCl, 10 mM MgCl_2_, 1 mM dithiothreitol (pH 7.9 @25°C); NEB4∶ 50 mM potassium acetate, 20 mM Tris-acetate, 10 mM magnesium acetate, 1 mM dithiothreitol (pH 7.9 @ 25°C).
